# Decomposition nitrogen is better retained than simulated deposition from mineral amendments in a temperate forest

**DOI:** 10.1111/gcb.13450

**Published:** 2016-08-23

**Authors:** Richard K. F. Nair, Michael P. Perks, Maurizio Mencuccini

**Affiliations:** ^1^ School of Geosciences University of Edinburgh Crew Building Edinburgh EH9 3FF Midlothian UK; ^2^ Forest Research Northern Research Station Roslin EH25 9SY Midlothian UK; ^3^ ICREA at CREAF, Campus de UAB Cerdanyola del Vallés Bellaterra, Barcelona Spain; ^4^ Present address: Max Planck Institut für Biogeochemie 07745 Jena Germany

**Keywords:** ^15^N‐nitrogen, forest, isotope trace, litter decomposition, litter nitrogen, nitrogen fertilization

## Abstract

Nitrogen (N) deposition (N_DEP_) drives forest carbon (C) sequestration but the size of this effect is still uncertain. In the field, an estimate of these effects can be obtained by applying mineral N fertilizers over the soil or forest canopy. A ^15^N label in the fertilizer can be then used to trace the movement of the added N into ecosystem pools and deduce a C effect. However, N recycling via litter decomposition provides most of the nutrition for trees, even under heavy N_DEP_ inputs. If this recycled litter nitrogen is retained in ecosystem pools differently to added mineral N, then estimates of the effects of N_DEP_ on the relative change in C (∆C/∆N) based on short‐term isotope‐labelled mineral fertilizer additions should be questioned. We used ^15^N labelled litter to track decomposed N in the soil system (litter, soils, microbes, and roots) over 18 months in a Sitka spruce plantation and directly compared the fate of this ^15^N to an equivalent amount in simulated N_DEP_ treatments. By the end of the experiment, three times as much ^15^N was retained in the O and A soil layers when N was derived from litter decomposition than from mineral N additions (60% and 20%, respectively), primarily because of increased recovery in the O layer. Roots expressed slightly more ^15^N tracer from litter decomposition than from simulated mineral N_DEP_ (7.5% and 4.5%) and compared to soil recovery, expressed proportionally more ^15^N in the A layer than the O layer, potentially indicating uptake of organic N from decomposition. These results suggest effects of N_DEP_ on forest ∆C/∆N may not be apparent from mineral ^15^N tracer experiments alone. Given the importance of N recycling, an important but underestimated effect of N_DEP_ is its influence on the rate of N release from litter.

## Introduction

Quantitative estimates of the effect of anthropogenic nitrogen deposition (N_DEP_) on temperate forest C uptake and sequestration can vary by an order of magnitude (de Vries *et al*., 2009). Some studies comparing regional N_DEP_ with indices of forest productivity or growth (Magnani *et al*., 2007; Thomas *et al*., 2009; Ferretti *et al*., 2014) report greater effects of N addition on C uptake (ΔC/ΔN) than estimates obtained from N budget or ^15^N‐tracer additions (Nadelhoffer *et al*., 1999; de Vries *et al*., 2009). These low estimates are based on evidence indicating low C : N sinks (e.g. microbial communities and immobilization in soil fractions) are more competitive than trees for mineral ^15^N (Templer *et al*., 2012). Only about ¼ of added ^15^N fertilizer obtained by trees is assigned to high C : N wood (Nadelhoffer *et al*., 1999). Consequently, process‐based models tend to represent soil immobilization of N as limiting tree N uptake (Gerber *et al*., 2010; Zaehle *et al*., 2010; Thomas *et al*., 2013) and similarly predict modest effects of N deposition on forest C uptake.

This difference in ΔC/ΔN among the studies above is usually attributed to covariance of N_DEP_ at the continent or country scale with other drivers of a growth response (de Schrijver *et al*., 2008; Sutton *et al*., 2008; de Vries *et al*., 2008) as while temperate and boreal regions are typically considered N‐limited (Vitousek & Howarth, 1991), many other global change drivers (Sedjo, 1992; Norby, 1999; Prentice *et al*., 2001; Saxe *et al*., 2002) vary over the geographic range of correlative studies. Relatively little attention has been paid to artefacts of isotope studies which may affect understanding of ecosystem level N effects. ^15^N tracer experiments are predominantly applications of isotope‐enriched mineral N fertilizers, for example ammonium nitrate, made periodically directly to the soil surface. These may raise total N inputs substantially above ambient levels of atmospheric deposition, especially if enrichment is low. Conversely, real‐world ambient N_DEP_ is of low intensity (Aber *et al*., 1998) and chronic (Lovett & Goodale, 2011), occurring over forest canopies in a variety of organic and inorganic forms. Additionally, even under high N_DEP_, N mineralized from litter recycling is usually greater than N added in deposition or fertilizer (Schulze, 2000; Högberg, 2012) or N fixation (Cleveland *et al*., 1999). N from litter sources is available continuously and is slowly depolymerized through many intermediate forms before becoming mineral ${\text{NH}}_{4}^{+}$ or ${\text{NO}}_{3}^{-}$_._ These organic products of litter are typically considered unavailable to plants before being fully mineralized.

In some situations, plants, or plant–mycorrhizal symbioses, can, however, take up organic N forms without initial reduction to ${\text{NH}}_{4}^{+}$ (Näsholm *et al*., 2009). Organic N can reach high concentration in soils and includes amino acids, peptides, and proteins (Schulten & Schnitzer, 1997). Bioavailability of organic N could increase N availability for trees, allowing more N to be obtained despite strong soil sinks for mineral ions N repeatedly demonstrated in mineral fertilization experiments. Some of these forms may be acquired by mycorrhizal symbionts (Leigh *et al*., 2009), and reduced before transfer to plants, while molecules as large as proteins may be utilized directly by roots in the laboratory (Paungfoo‐Lonhienne *et al*., 2008) without mycorrhizal or microbial assistance. In the field, dual ^13^C/^15^N labelling also demonstrates amino acids incorporated whole into temperate forest roots (Rothstein, 2014) as well as in high latitude forests where amino acids dominate N availability (Inselsbacher & Näsholm, 2012). Most evidence suggests that organic N uptake is most important under such conditions of limiting mineral N supply (Chapin *et al*., 1993; Näsholm *et al*., 1998; Schiller *et al*., 1998; Rennenberg *et al*., 2009). However, as older literature suggests that mineral N is the only ecologically relevant pool for N uptake, this process is also relatively understudied (Näsholm *et al*., 2009) so may be overlooked in other forest ecosystem studies. In forests, availability (and hence potential for uptake) of organic N may also depend on stand age and microbial community development, and organic N may be a substantial proportion of total N availability (Leduc & Rothstein, 2013). Uptake of N from heterogeneous organic sources such as microbial cells (Vadeboncoeur *et al*., 2015) and plant litter (Zeller *et al*., 2000; Guo *et al*., 2013a) has been demonstrated, although plant ^15^N recovery varies. Uptake of organic decomposition products may also be more energetically efficient (Zerihun *et al*., 1998; Gruffman *et al*., 2013) than incorporating mineral N and may affect structural development both above‐ and belowground (Gruffman *et al*., 2012), increasing the potential to alter overall C sequestered in woody tissues. Addition of mineral as opposed to organic forms of N also shows different effects on soil processes (Du *et al*., 2014), which may also mean N released from litter turnover has different effects on soil C and N cycling than mineral additions.

If decomposed N is better retained in soil or plants than mineral N, this would indicate mineral tracer‐based frameworks may underestimate ΔC/ΔN. As N inputs can affect litter decomposition rates both upward and downward (Knorr *et al*., 2005), mediating decomposer community structure (Frey *et al*., 2004), litter C/N ratios (McNulty *et al*., 1991) and interacting with litter quality and environmental drivers, mineral ‘N_DEP_’ treatments may also have effects on amounts of N released from decomposition and available in an organic form. Increases or decreases in this N released from litter decomposition may have different effects on N availability to both plants and soil biota than mineral N inputs.

Here, we combine an experiment replacing the litter layer with a unique source of ^15^N‐labelled litter, with a ‘deposition’ experiment where we apply a solution of ^15^N‐labelled NH_4_NO_3._ While wet‐applied NH_4_NO_3_ is neither necessarily representative of heterogeneous atmospheric N inputs, which are both wet and dry forms of N, nor of throughfall and stemflow N, which have passed through the canopy, it is consistent with the majority of N addition studies, which employ either NH_4_NO_3_ or either ion, usually directly to the soil. Hence, our applications are used to simulate typical N deposition treatments, rather than being strictly representative of N deposition itself.

Few studies (Zeller *et al*., 2000; Weatherall *et al*., 2006; Zeller & Dambrine, 2011; Hatton *et al*., 2012; Guo *et al*., 2013a,b) have used a ^15^N‐enriched litter source in the field to trace N from decomposition and we could not identify any work where the fate of ^15^N in deposition or added as fertilizer in the field is directly compared to ^15^N from litter release. Here, we use small N amendments in frequent dilute applications and our N fertilization treatments are similar to ambient N inputs and not intended to induce a N dosage treatment effect, while also close to expected N release from litter to minimize differences in patterns of ^15^N distribution due to different temporal patterns of N availability. Differences, if observed, are designed to be attributable to ^15^N source rather than differences in total ^15^N or N availability between treatments.

Our null hypotheses were that recovery of ^15^N from litter is the same as from conventional mineral ^15^N deposition‐simulating additions (henceforth ‘deposition’) in (1) soils, (2) tree roots, (3) other litter, and (4) soil microbial biomass (SMB). Identical recovery would imply that mineral ^15^N traces can all explain ecosystem N partitioning. We expected recovery of ^15^N to be greatest in the upper soil horizons as these were closest to the ^15^N‐enriched sources in soils and litter.

## Materials and methods

### Study site

We worked at Cloich forest, a managed Sitka spruce (*Picea sitchensis* (Bong. (Carr.))) plantation 34 km outside of Edinburgh, United Kingdom (55°42′N, 03°16′W). It was established in 1970 at 2500 stems per hectare (2 m intertree spacing), and the area used for our experiment was unthinned. Previous work at the site (Greens *et al*., 1995) removed some low‐level branches to improve access, which we repeated, removing all branches up to 1.5 m above the ground. Our plot is approximately 400 m above sea level, and the soil is a shallow peat overlaying Silurian Ordovician greywacke (Sheppard *et al*., 1995). There is no understory, and the litter (L horizon) is mostly acidic needles with a layer of partially decomposed litter (O horizon). In this study, we combined the fermentation fraction of the litter with the O horizon. Below, there is a thicker, dark‐coloured A horizon of organic dominated peaty topsoil, with a sharp divide before an orange‐brown B horizon. This study focused on the organic horizons (L, O and A). Due to ploughing at establishment, soils were approximately 30 cm deep on furrows and 45 cm deep on ridges, layer depths varying with microsite topography: litter (1–7 cm) and O (3–11 cm) layers being deeper in furrows than on ridges.

Local climate is typical of southern Scotland with annual minimum temperatures of −0.2 °C in December and maxima of 18.8 °C in July. Annual rainfall is 980 mm, which frequently falls as snow in the winter. Background nitrogen deposition is estimated to be 14–16 kg ha^−1^ yr^−1^. In the area we selected, average dbh was 21.5 ± 5.70 (SD) cm.

### ^15^N manipulation treatments

We obtained artificially produced Sitka spruce ‘litter’ (foliage and small twigs) with an elevated ^15^N/^14^N ratio from a whole‐canopy harvest of ^15^N‐labelled trees (Nair *et al*., 2014). This was separated from branches by drying until needles were shed and then mixed, keeping source trees separate. Mean N concentration by dry weight in this artificial litter was 1.2%, while C % was 51.0% (C/N ratio 34). Fresh litterfall at the study site had an average N concentration of 1.1% and C concentration of 47.1% (C/N ratio 47.5).

We established twelve rectangular plots, each containing a central tree within a grid of up to eight peripheral trees (a single tree was missing from the corner of some plots), with an edge of c. 4 m on each side. Each plot was randomly assigned to one of four (*n* = 3) treatments, as follows:

Two treatments (LIT and DEP) had the entire litter layer removed with a shovel in November 2012 and immediately replaced with dry ^15^N‐labelled litter (treatment: LIT) or dry unlabelled litter [0.366 atom % (0‰ *δ* ^15^N), treatment: DEP]. The unlabelled replacement litter (DEP) was litter previously removed from the site, dried to a similar dry weight as the labelled litter, and sorted to remove large twigs and other debris. For the LIT plots, we combined litter from three source trees per plot, selecting from the set of heterogeneously enriched source trees to minimize the difference in mean ^15^N concentration per plot while also minimizing litter mixing. Thus, the individual LIT plots had ^15^N concentrations of 1.53 atom %, 1.87 atom %, and 2.09 atom %, while there were no significant differences in mean C or N concentration among ^15^N‐labelled mixes. The total dry mass of the litter applied varied slightly: 23.0, 22.2, and 21.7 kg for the ^15^N litter and 29.81, 29.52, and 27.07 kg for the unlabelled.

### Litterbags

We established a concurrent litterbag experiment in April 2013 to estimate rates of ^15^N loss from the labelled litter plots without disturbing the main experiment, and to estimate movement of litter‐derived ^15^N to other litter within the litter pool, via spatial separation of unlabelled litter and ^15^N‐labelled litter. The litter in litterbags was obtained from two trees with the same source as the labelled litter in the main experiment, one of which was ^15^N labelled, while one was an unlabelled ‘control’ tree from the same site. Sixty litterbags filled with 2‐g oven dry litter [20 ^15^N‐labelled litter (*δ* ^15^N ~9000‰) and 40 natural abundance litter (*δ* ^15^N ~0‰)] were constructed from 1.1‐mm aperture polypropylene mesh and sealed with a hot glue gun, then buried in the litter layer of the three additional plots (labelled/unlabelled/unswapped litter) established simultaneously with the main experiment, to avoid disturbance caused by litterbag removal and replacement. A plot of labelled litter (~2400‰) received 20 natural abundance litterbags (Treatment: ‘high ^15^N litter’), an unlabelled plot received 20 (~9000‰) litterbags (Treatment: ‘high ^15^N litterbag’), and a control natural abundance plot received 20 natural abundance litterbags (Treatment: ‘natural abundance control’). Three litterbags were retrieved per plot on nine occasions between April 2013 and May 2014. The litter from litterbags was processed in the same way as sequential litter samples from the main experiment.

### Sampling strategy

On eight occasions [immediately before the first deposition treatment (January 2013) until 6 weeks after the last deposition treatment (May 2014)], we removed soil samples at three locations per plot (36 cores in total per date) using a 5.5‐cm‐diameter, 20‐cm‐deep soil auger. On three occasions, a larger corer (6.5 cm diameter) was used and masses were adjusted accordingly. Cores were removed by removing and bagging the surface litter layer, then driving the auger directly into the soil. The coring locations were determined by stratified random sampling, such that at least one ridge and one furrow were always sampled from each plot. Locations were reselected if the core location was within 5 cm of a previous core, or if the auger encountered an irremovable stone or other obstacle. The soil from the cores was separated on‐site into the O and A soil horizons and combined to give one composite sample per plot per date for each of the two soil horizons, except for the first three dates when only the O horizon was sampled. If the B horizon was encountered, this was discarded, with its depth recorded, to allow appropriate adjustment of volume. The soil samples were stored in a coolbox and transported back to the laboratory (approximately two hours from sampling time) then held overnight at 4 °C, or processed immediately.

### Processing and measurement

All soil cores were immediately weighed to establish field wet weight then allowed to equilibrate to ambient humidity at room temperature (rewetting if necessary to prevent drying), before sieving to pass through a 2‐mm mesh. From this <2 mm soil fraction, small needle and root debris were removed with tweezers. Subsamples (15–20 g) were weighed into stainless steel trays and then dried in a 80 °C oven overnight, until a stable mass was reached. After drying, the soil was reweighed and used to calculate the dry mass of the whole core, and a subsample was milled in a stainless steel capsule on a Retsch MM400 ball mill (Retsch Ltd, Hope, UK), until a fine powder was achieved, suitable for mass spectrometry.

The material that did not pass through the sieve was washed in deionized water, gently dried, and sorted to separate roots from stones and other debris. The total mass of dry roots from each set of three composite cores was recorded, and subsamples were ball milled. Litter samples were washed in deionized water to remove surface residues and dried overnight at 80 °C. These were then ball milled.

At the end of the experiment, a single‐point assessment of soil microbial biomass N and ^15^N concentration was also made. A 10‐g equivalent dry weight of wet soil from the <2 mm soil fraction was weighed into glass jars for fumigation. The fumigation samples were exposed to chloroform in a dark vacuum oven for 3 days, then extracted, while unfumigated controls were extracted immediately. To extract N, both fumigated and unfumigated samples were shaken for three hours with 50 ml 0.5 m K_2_SO_4_, then filtered through preleached Whatman no. 1 filter paper. The filtrate was freeze dried for 2 days to remove all water, and a small subsample (~10 mg) was analysed for C and N content on a Carlo Erba NA 2500 elemental analyser. The remaining filtrate was rehydrated with deionized water to deliver an appropriate amount of N for capture in an acid diffusion trap, and processed via the N diffusion technique (Stark & Hart, 1996) by adjusting the pH of the solutions with conc. NaOH, adding 0.4 g of Devarda's alloy, and trapping the solution N on a preprepared PTFE‐enclosed KHSO_4_‐infused paper disc.

Samples were analysed for ^14/15^N (all samples) and ^12/13^C (all samples apart from diffusion traps) on a SerCon Callisto CF‐IRMS Isotope Ratio Mass Spectrometer, along with samples of known isotope abundance and method blanks for the N diffusion discs. To calculate N and ^15^N in the traps, the method blank discs were subtracted from the sample diffusion trap N concentrations.

### Statistical analysis and mass balance

We modelled the change in *δ* ^15^N in O and A horizon roots and soil separately, with linear mixed effects models. We used treatment and date as fixed factors and plot as a random factor. A correlation structure was used to control for pseudoreplication among successive measurements of the same plots over time and a weighting structure was employed to allow the residuals to increase later in the experiment when cumulative ^15^N inputs and potential *δ* ^15^N were larger. All statistics were performed in r v 3.01 (R Core Team, 2013), and linear mixed effect models were run with the nlme package (Pinheiro *et al*., 2013) with residuals inspected using normal probability quantile plots (qqnorm). Subsequent post hoc Tukey HSD tests were performed with the general linear hypothesis (glht) in the multcomp package (Hothorn *et al*., 2008). We also calculated $R_{m}^{2}$ (Nakagawa & Schielzeth, 2013) in order to break down linear model *R* ^2^ into a component relating to the fixed effects we were interested in.

As dry masses of soil horizons and roots were highly variable and did not differ statistically among treatments, we used their average masses and N concentrations to calculate N pool sizes in the bulk soil, roots, litter, and microbial biomass as enrichment in all plots with a ^15^N source (LIT, DEP, DEPu) over CONTROL. The experiment was designed to be maintained in the long term, so we did not remove, dry, and weigh the litter layer at this point, mass instead being informed by the dry masses of litter removed at the start of the experiment.

^15^N‐tracer recovery was expressed as a % of total applied ^15^N_DEP_ (DEP, DEPu), or total ^15^N calculated to have been released from the ^15^N‐labelled litter (LIT). This latter calculation was based on litterbag mass loss and changes in litter N concentration; net litter ^15^N release was assumed equal to the change in ^15^N concentration of the litter N pool (in g) since the beginning of the experiment. Errors on these estimates were propagated using standard methods, from measurements and between replicates, through to a final ^15^N‐tracer recovery.

## Results

### ^15^N inputs in litter and decomposition treatments

**Figure 1:**
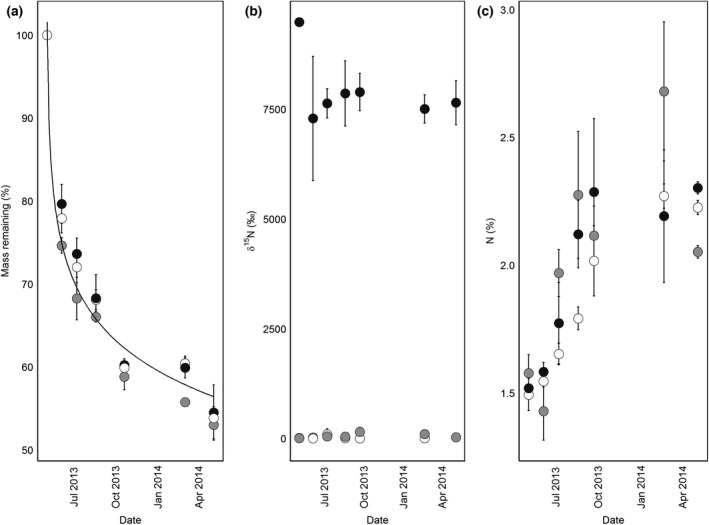
Decomposition in the litterbag experiment. Figures show (a) mass loss, (b) changes in ^15^N concentration, and (c) changes in N concentration over time. Treatments are as follows: unlabelled litterbag in unlabelled litter (white), unlabelled litterbag in ^15^N‐litter (grey), and ^15^N‐litterbag in unlabelled litter (black). Error bars show standard deviation.

**Figure 2:**
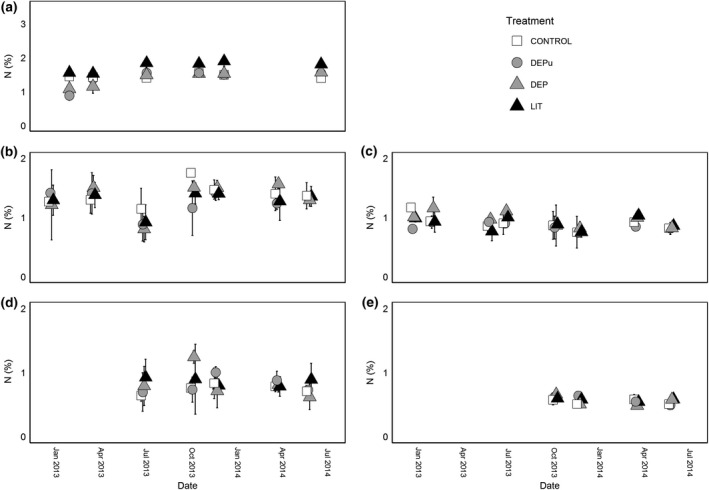
N (% by dry mass ± standard deviation) of forest floor (a: litter, b: O layer soil, c: O layer roots, d: A layer soil, e: A layer roots) pools over time. Treatments are as follows: CONTROL (white square), DEPu (grey circle), DEP (grey triangle), and LIT (black triangle) as described in Table 1. A small offset has been applied to the *x*‐axis to distinguish treatments.

We added a total of 1.18 g ^15^N per plot in the deposition treatments (DEP and DEPu) over the whole experiment. Over the year, the litterbags lost almost 50% of their mass (Fig. 1a), which fit a logarithmic curve (*R* ^2^ = 0.92), while N concentrations rose from 1.5% to ~2.25% (Fig. 1c). We used the litterbag change in mass, and observed changes in N concentration in litter in the main plots (Fig. 2, Table 1), to estimate that the litter layer mineralized a net ~32.5 kg N ha^−1^ yr^−1^. *δ* ^15^N stayed relatively constant in the high ^15^N litterbag treatment, while the unlabelled litterbags decomposing in the high ^15^N litter displayed some variance in *δ* ^15^N over time but did not significantly differ from the control litter (*P* > 0.05). Hence, in the main experiment, ^15^N released in LIT over natural abundance was 0.79–1.05 g per plot (varying due to litter source), close to the ~1.15 g ^15^N added in deposition to DEP and DEPu over the same time period.

**Table 1:** Summary of treatment combinations by levels of ^15^N‐enrichment of the litter layer and of the deposition

Treatment ID	Litter layer	Deposition
LIT	Swapped, ^15^N‐enriched	Natural abundance NH_4_NO_3_
DEP	Swapped, natural abundance	98% ^15^N – ^15^NH_4_^15^NO_3_
DEPu	Unswapped, natural abundance	98% ^15^N – ^15^NH_4_^15^NO_3_
CONTROL	Unswapped, natural abundance	Water

### N concentration and ^15^N expression in soil system pools over time

Soil system pools did not vary in N concentration over time, with no statistically significant differences among treatments in any of the five pools (O and A soil, O and A roots, and litter) over the treatment period (Fig. 2). In most pools, N concentration remained constant, except for the litter; here, average N concentrations were initially higher in the two swapped litter treatments (LIT and DEP), than the two unswapped treatments (DEPu and CONTROL) although this difference was quickly lost over time.

**Figure 3:**
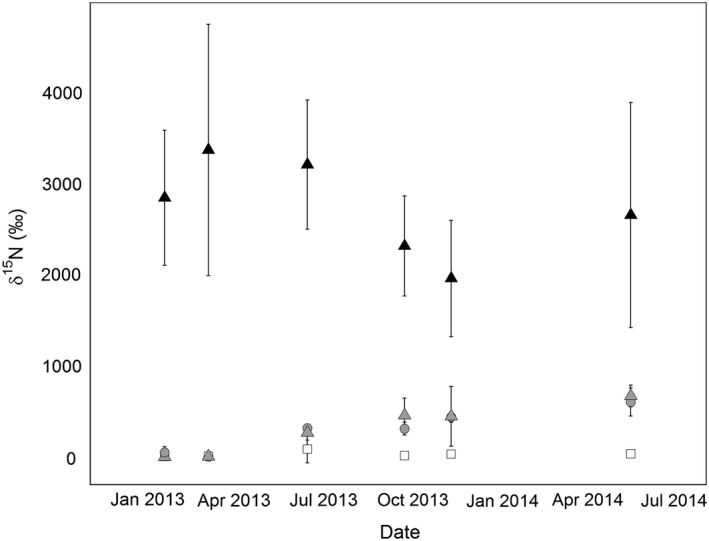
^15^N concentrations (*δ* ^15^N ± standard deviation) of the litter layer over time. Treatments are as follows: CONTROL (white square), DEPu (grey circle), DEP (grey triangle), and LIT (black triangle) as described in Table 1.

As ^15^N release from LIT and ^15^N added in DEP/DEPu was similar, we directly compared the *δ* ^15^N of soil horizons over time. The LIT litter layer was very highly ^15^N‐enriched (averaging around 2500‰) as this was the source of ^15^N enrichment. *δ* ^15^N fluctuated (Fig. 3) and variance was very high, which was expected as the litter mixes used for the swap were not completely homogeneous. Otherwise a consistent, but smaller increase was visible in litter *δ* ^15^N from the two labelled N_DEP_ treatments (Fig. 3) reaching a *δ* ^15^N in May 2014 of 670 ± 70‰ in DEP and 600 ± 90 in DEPu. When LIT treatment was removed from the data set to facilitate comparisons among the other treatments (which could be expected to have the same mean *δ* ^15^N if there was no effect of ^15^N treatments), Tukey HSD comparisons (Table 2) indicated that DEP and DEPu treatments were significantly (*P* = 0.004) different from CONTROL, but not from each other (*P* = 0.654).

**Table 2:** Tukey HSD comparisons for treatments in the most parsimonious model to explain litter layer *δ* ^15^N

Litter	DEPu	DEP	LIT
CONTROL	0.004**	0.004**	N/A
DEPu		0.654	N/A
DEP			N/A

Treatments are as described in Table 1. Asterisks indicate significance at the *P* < 0.05 (*), *P* < 0.01 (**), and *P* < 0.001 (***) level. Comparisons with LIT treatment were not made as this treatment did not have a null assumption of the same *δ* ^15^N as other treatments.

**Figure 4:**
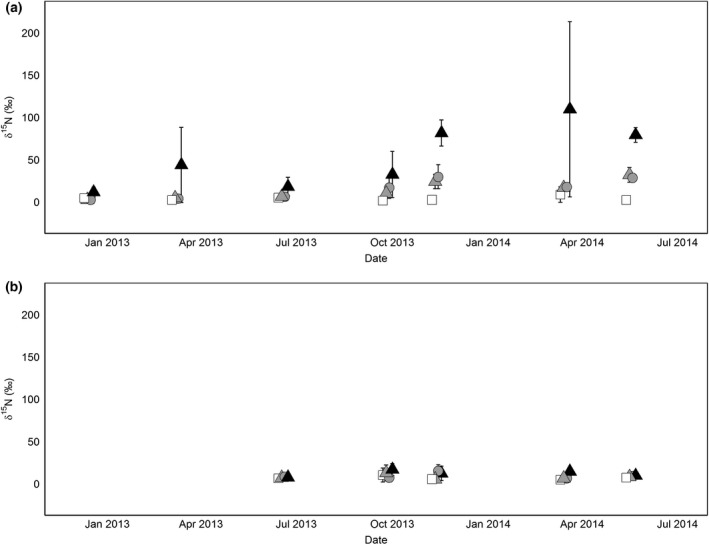
*δ* ^15^N (±standard deviation) of (a) O and (b) A soil layers over time. Treatments are as follows: CONTROL (white square), DEPu (grey circle), DEP (grey triangle), and LIT (black triangle) as described in Table 1. A small offset has been applied to the *x*‐axis to distinguish treatments.

In the O horizon soil, *δ* ^15^N increased in all ^15^N‐enriched treatments (Fig. 4), with the largest increases from LIT. In contrast, the DEP and DEPu had mean *δ* ^15^N slightly above natural abundance in the latter part of the experiment but remained similar to CONTROL (Table 3). By May 2014, the O soil had a *δ* ^15^N of 65.9 ± 13.6‰ (SD) in LIT, 29.5 ± 14.5‰ in DEP, 26.0 ± 6.9‰ in DEPu, and 2.2 ± 0.4‰ in CONTROL. Variance was large as our sample size was small. The linear relationship fit to these data revealed significant effects of both treatment (*P* = 0.002) and date (*P* < 0.001) on *δ* ^15^N in this horizon, due to contrasts between LIT and the other treatments (*post hoc* Tukey HSD). $R_{m}^{2}$ for this model indicated that fixed effects (treatment and date) accounted for 49 % of the variation.

**Table 3:** Tukey HSD comparisons for treatments in the most parsimonious model to explain O_h_ soil *δ* ^15^N

O_h_ horizon	DEPu	DEP	LIT
CONTROL	0.540	0.426	<0.001***
DEPu		0.997	0.004**
DEP			0.027*

Treatments are as described in Table 1. Asterisks indicate significance at the *P* < 0.05 (*), *P* < 0.01 (**), and *P* < 0.001 (***) level.

In contrast in the A horizon soil, there were no significant differences among any of the treatments (*P* = 0.065) and over time (*P* = 0.758) in *δ* ^15^N‰ (Fig. 4). *δ* ^15^N measured in the CONTROL A horizon was 6.5 ± 0.8‰, similar to unlabelled control treatments in other ^15^N‐N_DEP_ experiments (Nadelhoffer *et al*., 1995), and slightly more enriched than our O horizon fractions (3.6 ± 1.0‰).

### N concentration and ^15^N expression in roots over time

**Figure 5:**
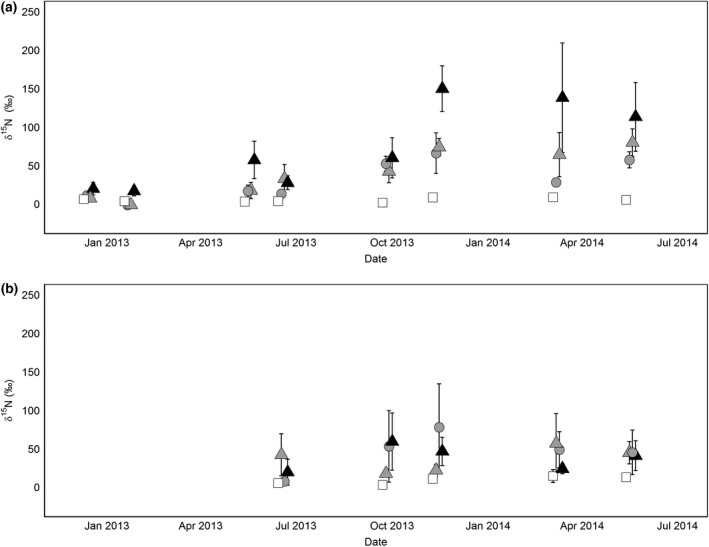
*δ* ^15^N (±standard deviation) of (a) O and (b) A soil layer roots over time. Treatments are as follows: CONTROL (white square), DEPu (grey circle), DEP (grey triangle), and LIT (black triangle) as described in Table 1. A small offset has been applied to the *x*‐axis to distinguish treatments.

Like the soil, *δ* ^15^N also increased in the roots (Fig. 5). In the O horizon, the treatment ^15^N increased, reaching maxima of LIT 149.7 ± 29‰ (SD) DEP 79.7 ± 18‰, and DEPu 65.9 ± 26‰. The mixed effect model for this horizon had a significant effect of date (*P* = 0.036), treatment (*P* < 0.001) and their interaction (*P* < 0.001) which overall explained 69% ($R_{m}^{2}$) of the variation (Table 4). All treatments were significantly (Tukey HSD) different than CONTROL, and LIT was significantly different from all other treatments, although DEP and DEPu were not significantly different from each other.

**Table 4:** Tukey HSD comparisons for treatments in the most parsimonious model to explain root *δ* ^15^N in both soil layers

	DEPu	DEP	LIT
O_h_ horizon			
CONTROL	<0.001***	<0.001***	<0.001***
DEPu		0.977	0.007**
DEP			0.018*
A_h_ horizon			
CONTROL	0.033*	<0.001***	0.015*
DEPu		0.581	0.998
DEP			0.756

Treatments are as described in Table 1. Asterisks indicate significance at the *P* < 0.05 (*), *P* < 0.01 (**) and *P* < 0.001 (***) level.

For the A horizon, the regression showed significant differences, both for treatment (*P* < 0.01) and over time (*P* < 0.001), with LIT, DEP, and DEPu being all significantly different from CONTROL but not from each other (Table 4). *δ* ^15^N in the roots of this horizon was higher in the ^15^N‐enriched treatments than CONTROL but tended to be below 50‰ (Fig. 5). There was no significant interaction between date and treatment in the most parsimonious model. $R_{m}^{2}$ for this was lower, explaining only 25% of the variation.

### K_2_SO_4_ extractable ^15^N and microbial ^15^N return

There were no significant differences in extractable N among the four treatments and two soil layers (*P* > 0.05) in the May 2014 harvest. The mean N extractable was 0.024 ± 0.03 (SD) mg g^−1^ in the O layer, and 0.010 ± 0.01 mg g^−1^ in the A layer. *δ* ^15^N of the horizon O_*h*_ extract was significantly greater in LIT, DEP, and DEPu (combined mean = 171.2 ± 40) than CONTROL (mean 66.3 ± 8, *P* = 0.004) but these were not different from each other (data not shown). There were no significant differences in the A layer extractable N *δ* ^15^N.

We did not apply a correction factor for microbial nitrogen. The difference in N extracted between fumigated and unfumigated samples (indicative of microbial N) was 0.092 ± 0.06 (SD) mg g^−1^ in the O horizon and 0.043 ± 0.04 (SD) mg g^−1^ in the A horizon. Mean *δ* ^15^N of the O microbial biomass was significantly higher in LIT, DEP, and DEPu (overall mean = 171.8 ± 140‰) than CONTROL (29.2 ± 9‰, *P* = 0.02) but not different among treatments. There were no significant differences in *δ* ^15^N of this pool in the A_*h*_ horizon, where the control *δ* ^15^N was 47.4 ± 13‰.

### Mass balance estimates of soil ^15^N return

We could account for most of the ^15^N available in the soil system in both LIT and DEP in the endpoint mass balance (Table 5), although propagated errors tended to be high due to the high number of uncertain quantities and small sample size. Most ^15^N recovery was in the litter horizon, where we recovered ~80–90% of N in DEP and DEPu. A similar assessment was not available for LIT; we intended to use the litterbags to estimate litter–litter transfer of N but had no returns in this pool (see Discussion) so we used mineral ^15^N‐addition retention in litter to estimate retention of litter ^15^N in the litter layer for the mass balance.

**Table 5:** Mean mass and N%, and (below the line) mean percent mass balance ^15^N balance recovery of ^15^N tracer in May 2014 in treatments DEPu, DEP, and LIT as enrichment above CONTROL (see Table 1 for definitions)

	Litter	O horizon			A horizon		
		Roots	Soil	Microbes	Roots	Soil	Microbes
Mass (kg ha^−1^)	3800_a_	8100 (3000)	61 600 (1400)	5.70_b_ (1)	7800 (2000)	174 800 (31 000)	7.70_b_ (1.1)
N (%)	1.58 (0.3)	0.89 (0.1)	1.35 (0.4)		0.66 (0.1)	0.81 (0.3)	
DEPu	83.58_a_ (48.08)	2.44 (1.6)	15.78 (10.9)	0.59 (0.17)	1.05 (0.7)	0.66 (2.0)	0.13_c_ (0.11)
DEP	90.98_a_ (51.97)	3.49 (2.3)	13.87 (9.5)	0.96 (0.23)	1.06 (0.8)	1.59 (2.9)	−0.04_c_ (0.07)
LIT	NA_a_	6.42 (3.0)	50.71 (24.4)	1.48 (0.48)	1.06 (0.5)	2.19 (5.9)	−0.10_c_ (0.06)

Values show standard errors of the mean in parentheses. Subscripted values indicate the following: (a) estimates were obtained using litter pool masses (which may be overestimates). (b) Microbial N is a proportion of the measured soil pool and should not be included as a separate component of the total. This figure is not adjusted by a correction factor for total microbial biomass so N % is also not presented. (c) In some cases, the A layer microbial biomass was on average ^15^N depleted relative to the control and hence a negative tracer recovery.

With litter excluded, total system recovery was 60.39% from LIT, and 20.12% from DEP and DEPu together, despite the slightly larger total ^15^N inputs in the DEP treatments. N recovery was highest in the O horizon; here in the LIT treatment, we calculated a recovery of ~50% of ^15^N released from the litter, compared to 14% of ^15^N from inputs in DEP. Very little ^15^N was found in the A horizon, being 1–3% of ^15^N available in all treatments and standard errors in this treatment were greater than the mean.

Similarly, root recovery of ^15^N was higher in the O horizon. Because the mass of the root pool was relatively smaller than soil, the high *δ* ^15^N observed accounted for only ~3.5% in DEP and ~6.5% in LIT. Root ^15^N return in the A horizon was about 1% of the total N in all treatments.

Only small proportions of the soil ^15^N recovery were derived from microbial biomass in all treatments which accounted for around 0.5–1.5% of ^15^N in the O horizon and none of the ^15^N in the A horizon. As total extracted N from the soils was low and already included in total soil ^15^N return, we did not include K_2_SO_4_ extractable ^15^N in our mass balance calculations. Overall, the litter (LIT)‐derived ^15^N appeared to be retained in the soil around three times as much as deposition (DEP) ^15^N. When litter was included, close to 100% of DEP was estimated to have been recovered from the system.

## Discussion

Overall, we found greater soil system retention of N released from litter (LIT) compared to new N inputs from mineral N additions (DEP). Excluding the litter, where most DEP recovery was found, but intralitter ^15^N transfer was uncertain, around 60% of litter decomposition ^15^N was recovered in the soil, while 20% of ^15^N added as mineral fertilizer was recovered in the same pools (Table 5). Most of this difference was in the organic (O) horizon, and tracer recovery decreased with soil depth. However, there was proportionally greater ^15^N expression in roots, compared to soil. As total N additions were near‐identical between ‘deposition’ and litter treatments, and total ^15^N availability was similar, N from the litter source was substantially better retained than the fertilizer additions.

### Representativeness of litter and ‘deposition’ simulation

An important caveat to interpreting our results is whether our ‘DEP’ treatment faithfully represented nitrogen deposition, and whether our litter swaps provided a realistic litter layer. For the latter, there were no differences between disturbed (DEP) and undisturbed (DEPu) litter with N additions, indicating that ^15^N recovery was driven by ^15^N source (simulated deposition or litter) not an effect of the litter swap. However, our ‘deposition’ inputs differ from atmospheric inputs, which contain other compounds, are deposited chronically in both wet and dry forms, and are intercepted by the canopy before reaching the soil. Our low concentration, frequent NH_4_NO_3_ additions matched as best possible chronic deposition. And ammonium and nitrate are commonly used as a proxy for N deposition reaching the soil in field experiments, particularly when a ^15^N fertilizer is used (e.g. Tietema *et al*., 1998; Yao *et al*., 2011). Dry deposition inputs are typically not simulated due to the logistical complications involved, and in many cases, the magnitude and chemical composition of these background inputs is badly documented and variable. N deposition experiments also commonly assume an instantaneous mixing of inputs into soil pools when in ambient conditions movement of dry deposition depends on subsequent rainwater inputs. As such, ^15^N‐partitioning from our ‘DEP’ treatments is representative of common ^15^N experimental methodology, and many of the caveats relevant to interpreting this directly as N deposition partitioning also apply here.

### Depth‐dependent ^15^N recovery

In both our deposition‐simulating and labelled litter treatments, most ^15^N recovered was found in the litter (Fig. 3) and O layer soil (Fig. 4), where more ^15^N was recovered from the litter source (50%) than the mineral inputs (13–15%). Summed, and excluding the high litter recovery, mineral N recovery in soil was lower than most fertilization studies (Templer *et al*., 2012), but similar to recovery in studies of low N additions (Koopmans *et al*., 1996; Micks *et al*., 2004) and similarly biased towards upper (organic) soil. Total soil and root ^15^N recovery from the A horizon were lower (1–3% of total ^15^N released) and correspond to low recovery in this horizon from the majority of forest ^15^N‐fertilizer studies (Nadelhoffer *et al*., 1999; Templer *et al*., 2012).

Most of the ^15^N recovery in LIT (around three times the recovery of DEP) was also in the O horizon, but similarly low in the A horizon. Some litter ^15^N can be recovered in deeper soil layers in long‐term trace experiments (Eickenscheidt & Brumme, 2012), but sinks in upper soil layers predominate, and our results indicate that ^15^N released from litter was more resistant to leaching down through the soil profile. Some of this ^15^N decomposed from litter may have remained in the litter layer, but litter–litter ^15^N transfer was not found in our litterbag experiment. If it is assumed that this is an artefact, and litter ^15^N was similarly retained within the litter layer as in DEP (discussed in the litter decomposition section), we can account for more than 100% of the released label. Even if litter retains less decomposed ^15^N, this is still substantially more than inferred from DEP, implying an overall greater recovery of the tracer in the soil system.

Most of this extra litter source N is probably in organic forms (Warren, 2014) but not all forms of organic N are likely equally bioavailable, if at all. Larger molecules are unlikely to be accessible, but also less mobile in soil than mineral ions (particularly ${\text{NO}}_{3}^{-}$), and less vulnerable to gaseous losses via denitrification or leaching (Butterbach‐Bahl *et al*., 2011). Further decomposition of this N may be gradual, slowly releasing N into plant‐available forms, such as amino acids. These are most chemically similar to NH_3_ and may dominate N uptake in boreal zones, while less is known about their importance in temperate regions where mineral forms of N are more available. Amino acid ^15^N addition recovery in soils is typically not different than mineral ^15^N additions, unlike recovery in plant tissues (Näsholm *et al*., 2009; McFarland *et al*., 2010), so in LIT, the presence of larger ^15^N‐enriched products of decomposition explains the high O horizon ^15^N recovery. We could find no studies on recovery of additions of larger ^15^N‐labelled polymers in the field to understand how representative ~50% ^15^N recovery in this horizon may be. Additionally, in our time series (Fig. 4), it is not clear if the increasing (variable) recovery in the soil only develops after October 2013. This could indicate release of these less mobile products at this time but not earlier during the litter mass loss (Fig. 1a). Earlier losses of ^15^N from the litter are visible from increasing root ^15^N early in the experiment (Fig. 5) so this difference may be due to sequential release of N‐compounds from decomposition.

### Litter decomposition and litter layer recovery of ^15^N‐nitrogen

Including litter, our total 80–90% recovery of the mineral‐applied isotope treatments is higher than most literature, although uncertainty is large due to small sample size and variability in biomass pool estimates. Recovery of mineral ^15^N in litter is variable but can be around 50% of added ^15^N (Downs *et al*., 1996) as decomposers assimilate N for the early stages of litter decomposition (Parton *et al*., 2007), litter having a higher C/N ratio than decomposer organism. Our higher than usual recovery may be due to frequently supplying the N sink in the litter layer with small inputs of ^15^N while never saturating N demand. Rather than variation in *δ* ^15^N of fresh litterfall (which is a few parts per million, Weber *et al*., 2008), variability in litter layer *δ* ^15^N (Fig. 3) probably reflects differences in decomposition rates, or decomposer colonization across the plot (Wang *et al*., 2013) which our small sample size would be unlikely to capture at any single time point. Stand establishment meant that litter depths varied substantially on ridges and in furrows, which may cause variation in thermal properties (Ogée & Brunet, 2002) and water retention (Putuhena & Cordery, 1996) across microsites.

Similarly, in LIT, litter *δ* ^15^N did not change over time (Fig. 3) but was highly variable, indicating a great deal of heterogeneity in ^15^N expression. Decomposition and variation in these rates across the plot could raise ^15^N concentrations due to fractionation (Kramer *et al*., 2003), but *δ* ^15^N variance was also likely due to insufficient mixing of the labelled litter at the start of the experiment. Litter mixing was carried out to control for factors which would affect ^15^N release from the litter across the plots, including differences in *δ* ^15^N of the source canopies (Nair *et al*., 2014), and litter quality between trees (Knorr *et al*., 2005; Berg & McClaugherty, 2008). Such a difference was evident early, where mass change differed between litterbag treatments (Fig. 1a), reflecting early loss of nonstructural C and acid‐hydrolysable materials (Berg, 2000) in fresh litter that had not naturally senesced (Chapin *et al*., 1990, 1993).

From these litterbags, we also did not detect any litter to litter ^15^N transfer. Tracer exchange between litters (Schimel & Hättenschwiler, 2007; Berglund *et al*., 2013) may only be possible in litter mixes when distinct components [e.g. mixed‐species litters, Berglund *et al*. (2013)] can be identified without physical separation imposed by litterbags. Thus, the lack of recovery of litter‐derived N in unlabelled litter may be an artefact of design and some litter ^15^N lost from decomposing litter was likely subsequently reincorporated by colonizing decomposers. If we assume a similar (80–90%) recapture of litter‐derived N in litter to DEP ^15^N additions, LIT recovery is more than 100% of the litter‐applied label. Deposition treatments were applied to the litter surface and percolated through the entire litter layer, while organic decomposition products are released throughout this horizon, so more DEP‐^15^N than LIT‐^15^N may be incorporated into litter but it is not clear how much this differs.

### Microbial recovery of tracer

Apart from litter and soils, microbes are major assimilators of mineral N additions over the short term (Jackson *et al*., 1989; Zak *et al*., 1990; Zogg *et al*., 2000; Morier *et al*., 2012) but recovery rapidly declines over the longer term (Zogg *et al*., 2000; Providoli *et al*., 2006; Templer *et al*., 2012) due to rapid pool turnover. Most of the soil recovery in both our mineral and litter ^15^N treatments was not found in microbes at the end of the experiment (some 2–3% ^15^N in O in all three treatments, and lower in A). Much of the ^15^N added earlier in the experiment may have been processed by this pool and be found elsewhere by the end of the experiment. We did not apply a correction factor for extraction efficiency, as little literature is available to obtain appropriate values for forest soils at 0.5 m K_2_SO_4_. Applying a similar 0.54 K_EN_ as in (Brookes *et al*., 1985) would indicate microbial ^15^N return almost two times larger and suggest a larger absolute difference in microbial return among treatments, although still a small proportion of total amendments.

### Potential losses

We can interpret differences in ‘missing’ ^15^N as ^15^N moved aboveground by root uptake if we can discount potential losses due to leeching and trace gases. Our design did not measure these losses, but leachate losses commonly amount to <10% of added mineral N from low additions of ^15^N fertilizer (Tietema *et al*., 1998; Zak *et al*., 2004; Providoli *et al*., 2005). The acidic soils at our site may have increased these losses due to their ion retention capacity, although the overall high recovery of tracer (80–90%) suggests that magnitude of N inputs and losses via leaching were not higher than usual. ^15^N losses as gases (such as NO_x_) from N_DEP_ are also rarely quantified (Templer *et al*., 2012), although likely to be low (Tietema *et al*., 1998; Christenson *et al*., 2002). For litter‐^15^N additions, organic N in leachate and tracing of losses of litter ^15^N via soil water have not been measured in many other labelled litter studies (Zeller & Colin‐Belgrand, 2001; Blumfield & Xu, 2004; Weatherall *et al*., 2006) but Eickenscheidt & Brumme (2012) found around 1% of ^15^N from labelled beech litter was lost as N_2_O over 10 years. Hence, for both DEP and LIT, ^15^N lost by these pathways is also likely to be minimal, and in both DEP and LIT treatments, the N cycle likely remains closed.

### Root recovery of tracer and implications for whole tree nutrition

Any ‘extra’ decomposition N found in the soil system is important for additional primary productivity and C uptake only if it is also obtained and distributed within plants. Around 20% of deposition treatment ^15^N (Nadelhoffer *et al*., 1999; Templer *et al*., 2012) is typically found in trees, which is plausible in our experiment but potentially obscured by high errors on soil pools. Our root recovery of ^15^N (Fig. 4) corroborated such findings; we found similar ^15^N recovery (~4.5% in total, Table 5) in DEP to other mineral N addition studies (c.f. Nadelhoffer *et al*., 1999bb; Templer *et al*., 2005) and around three‐quarters of ^15^N acquired is moved aboveground and expressed in aboveground tissues (Templer *et al*., 2012) and thus not represented in belowground recoveries. However, when our ^15^N tracer was from decomposition (LIT), root recovery (~8.5%) was on average almost double that in DEP. Hence, relative to total availability, more recycled litter N may be obtained by plants than when added in mineral fertilizers. While we did not measure aboveground pools (due to the large standing biomass and consequent isotope dilution effect), evidence for a proportionally greater whole tree recovery can be found in the roots as proportionally more litter ^15^N recovery was found in the A horizon roots (1%) than A horizon soil (6.5%) compared to the O horizon roots (2%) and O horizon soil (50%). This was despite the greater soil ^15^N recovery in the O horizon. ^15^N expression in deeper roots indicates translocation of ^15^N within the plant following uptake in the O horizon and may be reflected in other aboveground tissues.

Even with isotope techniques, it is difficult to quantify plant uptake of organic N as tracer recovery is insensitive to the form in which N is obtained, and N may be mineralized before uptake. Dual ^13^C and ^15^N‐labelling can address this problem, but this is not without difficulty in interpretation (Jones *et al*., 2005) and it was not possible to label the litter created for this study with ^13^C. Observed ^15^N enrichment in roots could be due to uptake of organic ^15^N or an overall more sustained mineral availability as organic N is decomposed continuously rather than added in distinct pulses. We tried to limit these differences by applying high frequency, low doses of ^15^N fertilizer in DEP/DEPu treatments, although this was monthly and to the soil surface and not continuous from the litter. However, K_2_SO_4_‐extractable ^15^N did not differ, so labile ^15^N was similar between DEP and LIT 6 weeks after the last application of the mineral tracer, indicating that variation in ^15^N availability to plants due to infrequent fertilizer use was minimal.

In addition to this evidence for greater nutrition from litter N due to ^15^N recovery, the lack of litter–litter transfer in the litterbags (Fig. 1b) could indicate, instead of the artefact previously discussed, that all decomposed litter ^15^N left the litter layer and was leached deeper into the soil, lost as trace gases, or moved into aboveground portions of the tree. If this ‘missing’ (40%) N is in the tree, then contribution of litter ^15^N to plant nutrition is beyond what is implied by root recovery. We took the most conservative approach and assumed that this missing litter‐derived ^15^N was in the litter, but not measurable in our litterbag experiment, although this may underestimate its importance relative to mineral N. Assessing the importance of litter ^15^N to aboveground growth is critical for future work in this area.

### Comparing nitrogen fate from litter and from atmospheric deposition

So how important is uptake of N from decomposition compared to deposition (or deposition‐simulating fertilizer experiments)? Biomass growth requires N but different N sources and forms may differ in their importance for tree N nutrition between ecosystems and N availability gradients. As knowledge for models of the global effects of N deposition on forest growth and function are based on processes measured in experiments, understanding the difference between ecosystem partitioning of mineral fertilizers (usually used to describe N uptake) and root uptake of recycled organic N is necessary to predict the effect of N deposition which may affect rates of N release from litter.

In this study, we showed that in a temperate forest, N released from an isotopically distinct litter substitute is both better retained in ecosystems and partitioned differently among litter, soils, and roots when compared to the mineral N additions typically used to simulate N_DEP_. Our mineral additions produced results similar to the wide body of literature using ^15^N fertilizers for N tracing, while higher soil retention of litter‐^15^N was paired with partitioning favouring reacquisition of litter N by trees. Therefore, the effect of N_DEP_ on forest growth and C sequestration potential may also depend on the effect of extra N inputs on litter quantity, quality, and subsequent rates N release from litter, as well as the frequently measured short‐term partitioning of mineral N within ecosystems. Similarly, there is a lack of knowledge of rate‐dependent effects of N additions, and the degree to which ${\text{NH}}_{4}^{+}$ and ${\text{NO}}_{3}^{-}$ added as fertilizer treatments reflect not only N‐compounds released from decomposition but also all atmospheric inputs, for example dry deposition. Litter decomposition releases N continuously and most N ‘deposition’ treatments apply fertilizer N/^15^N tracers in large pulse events cumulative with and in excess of ambient N deposition. A fuller understanding of the fate of litter‐decomposed N is critical for predicting the effect of nitrogen additions on forest C uptake.
